# A New Immunofluorescence Assay Allows the Sensitive Detection of Anti‐Cytosolic 5′‐Nucleotidase 1A Autoantibodies

**DOI:** 10.1002/eji.70181

**Published:** 2026-03-29

**Authors:** Fleur N. Brinkman, Isa G. A. Verlangen, Filine Swets, Ger J. M. Pruijn

**Affiliations:** ^1^ Department of Biomolecular Chemistry Institute for Molecules and Materials Radboud University Nijmegen the Netherlands

## Abstract

Anti‐cN1A autoantibodies are helpful for diagnosing inclusion body myositis (IBM). We have developed an immunofluorescence assay based on the perinuclear accumulation of GFP‐cN1A. The analysis of patient sera indicated that this assay shows a higher sensitivity for anti‐cN1A in IBM compared to alternative assays, without loss of specificity.

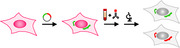

Inclusion body myositis (IBM) is a progressive idiopathic inflammatory myopathy, mostly occurring in men over 50 years old. IBM is characterized by inflammatory as well as degenerative features in affected patients’ muscles [1]. We and others have identified autoantibodies targeting cytosolic 5′‐nucleotidase 1A (cN1A; NT5C1A) and showed that these are frequently present in IBM patient sera [2, 3], and not or much less frequently in patients with other (autoimmune) diseases and healthy individuals [2, 3, 4, 5, 6].

Several tests have been developed to detect anti‐cN1A autoantibodies in patient sera. Virtually all of these assays are based on the immobilization of recombinant human cN1A protein or cN1A‐derived synthetic peptides, followed by incubation with patient sera and detection of bound antibodies with a labeled secondary antibody [7]. Studies reporting high specificities with large cohorts generally show a sensitivity of 33%–39% for IBM. The observation that the expression of cN1A in cultured human cell lines resulted in cN1A accumulation in perinuclear filaments prompted us to explore the applicability of these transfected cells for anti‐cN1A detection. We investigated whether transiently transfected HEp‐2 cells expressing the GFP‐cN1A fusion protein can be used for the detection of anti‐cN1A autoantibodies in autoimmune patient sera.

The typical accumulation of GFP‐cN1A in perinuclear filaments is illustrated in Figure 1A. The presence of both GFP and cN1A in the same subcellular structures was demonstrated by the colocalization of GFP fluorescence and staining of cN1A using a polyclonal anti‐cN1A antibody (Figure 1B). The integrity of the GFP‐cN1A fusion protein was substantiated by immunoblotting using the same anti‐cN1A antibody and a monoclonal antibody to detect GFP (Figure 1C). Incubation of fixed GFP‐cN1A expressing cells with anti‐cN1A positive and negative IBM patient sera, followed by a fluorescent secondary antibody, indeed resulted in anti‐cN1A‐dependent staining of the GFP‐cN1A accumulations (Figure 1D). Because previous studies reported anti‐cN1A autoantibodies of the IgA, IgG, and IgM isotypes [4, 8], we used a secondary antibody reactive with all of these isotypes. The direct comparison of the GFP and patient serum staining patterns allows the specific detection of anti‐cN1A autoantibodies, even in the presence of other autoantibodies reactive with antigens present in all the cells. Anti‐cN1A autoantibody staining is easily distinguishable from the staining patterns of other autoantibody reactivities.

**FIGURE 1:**
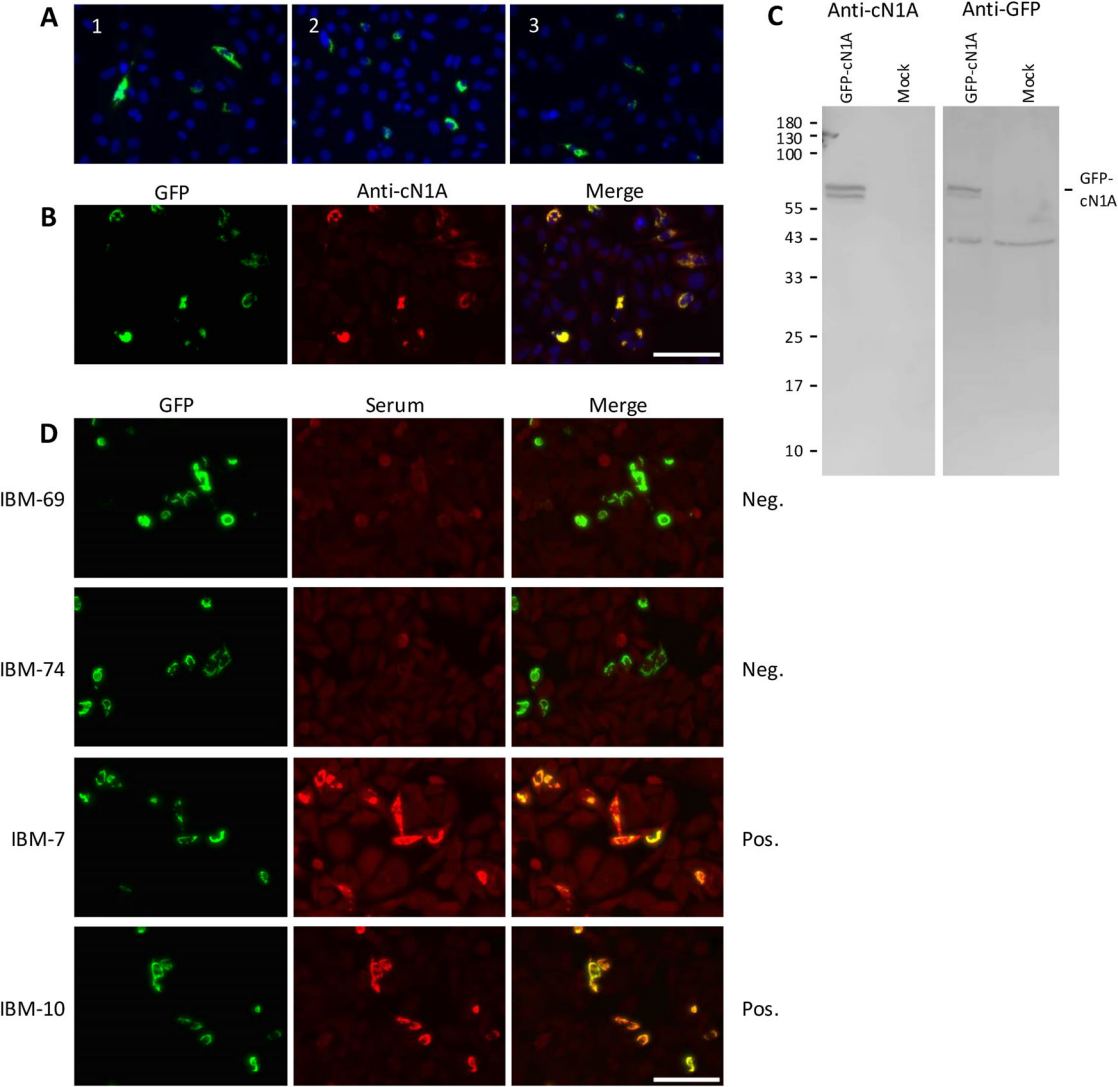
Reactivity of IBM patient sera with GFP‐cN1A expressed in HEp‐2 cells. (A) GFP‐cN1A fusion protein in transfected HEp‐2 cells visualized by fluorescence microscopy. Three representative images are shown (1‐3). Green: GFP‐cN1A; blue: nuclei stained by DAPI. (B) GFP‐cN1A expressing cells were also stained with a rabbit polyclonal anti‐cN1A antibody (red). (C) Western blot with lysates of GFP‐cN1A‐expressing and mock‐transfected HEp‐2 cells. Left: protein molecular weight markers (in kDa). Blots were stained with anti‐cN1A (left) and anti‐GFP (right) antibodies. (D) Transfected HEp‐2 cells stained with two anti‐cN1A‐negative and two anti‐cN1A‐positive IBM patient sera (red). Scale bar: 100 µm.

To assess the detection of anti‐cN1A reactivity in patient sera by this immunofluorescence assay, we first analyzed samples from 82 IBM patients. For every patient sample, two images of non‐overlapping areas were made, and three evaluators independently assessed these images. Forty eight of the 82 IBM patients (59%) appeared to be positive. Since anti‐cN1A autoantibodies have also been reported to occur, albeit at lower frequencies, in the sera from polymyositis (PM) and dermatomyositis (DM) patients, as well as in the sera from systemic lupus erythematosus (SLE) and Sjögren disease (SjD) patients, we next analyzed such patient sera (PM, *n* = 60; DM, *n* = 34; SLE, *n* = 28; SjD, *n* = 19), as well as samples from a group of healthy individuals (NHS, *n* = 35). Representative results for an anti‐cN1A‐positive patient from each of these diseases are shown in Figure S1. Seven out of 60 PM (12%) samples were anti‐cN1A‐positive, whereas only a single serum appeared to be positive for DM (3%), SLE (4%), and SjD (5%) (Table 1). None of the NHS sera showed anti‐cN1A autoreactivity in the immunofluorescence assay. This results in a sensitivity of 59% and a specificity of 93% based on disease controls only and 94% based on disease and healthy controls for anti‐cN1A autoantibody detection by immunofluorescence using GFP‐cN1A expressing cells.

**TABLE 1:** Anti‐cN1A reactivity in autoimmune patient sera determined by ELISA and immunofluorescence.

		Peptide ELISA^a^		Full length ELISA		Immuno‐fluorescence	
	Total (*n*)	*n*	%	*n*	%	*n*	%
IBM	82	31	38	31	38	48	59
DM	34	1	3	3	9	1	3
PM	60	4	7	8	13	7	12
SLE	28	7	25	0	0	1	4
SjD	19	7	37	1	5	1	5
NHS	35	0	0	0	0	0	0
Sensitivity			38		38		59
Specificity^b^			87		91		93

^a^ Data from Herbert et al. [5].

^b^ Based on non‐IBM disease samples.

The serum samples tested in the immunofluorescence assay were previously analyzed in an ELISA using three cN1A‐derived synthetic peptides representing three major linear epitopes of cN1A [5]. We also analyzed these sera in an ELISA with the full‐length recombinant human cN1A protein (produced in HEK293 cells). The results obtained with the synthetic peptide ELISA, the full‐length cN1A ELISA, and the immunofluorescence assay are summarized in Table 1. This comparison shows that the fraction of anti‐cN1A‐positive IBM patients is higher in the immunofluorescence assay (59%) than in both ELISA assays (peptide ELISA: 38%; full‐length protein ELISA: 38%). Interestingly, the number of anti‐cN1A‐positive PM and DM samples was higher in the full‐length protein assays (ELISA; immunofluorescence) than in the peptide ELISA, whereas the number of positive SLE and SjD samples in these assays was lower than in the peptide ELISA, although these results should be interpreted with caution because the number of positive samples was low (Figure S2). When all the non‐IBM patient samples are combined, similar specificities were obtained for the three assays, indicating that the increased sensitivity of the immunofluorescence assay is not associated with lower specificity.

To assess potential quantitative differences in anti‐cN1A levels between disease groups, titers were determined for all anti‐cN1A‐positive sera in the immunofluorescence assay. GFP‐cN1A‐expressing cells were incubated with serial serum dilutions (1:50 to 1:204,800), and the resulting images were blindly and independently assessed by three evaluators to determine the highest dilution that still resulted in positive anti‐cN1A signals. The results suggested that there are no major anti‐cN1A titer differences between IBM patients and patients suffering from other autoimmune diseases (Figure S3). We should, however, stress that the number of anti‐cN1A positive non‐IBM patients was rather low. The wide range of anti‐cN1A titers in IBM sera is in agreement with previous observations made by Goyal and coworkers, who reported titers ranging from 2000 to 100,000 when analyzed in a recombinant human cN1A ELISA [9].

The higher sensitivity of the immunofluorescence assay compared to both ELISAs is most likely due to antigenic differences. In the immunofluorescence assay, the full‐length cN1A protein is expressed in a human cell. Autoepitopes may be formed by proper folding of the protein, by the accumulation of cN1A in filamentous structures, and/or by post‐translational modifications. In the synthetic peptide ELISA, linear, small immunodominant epitope regions were used [5], which may not correspond to the autoepitopes displayed in cells. In the full‐length protein ELISA, conformational/modification‐dependent epitopes may also be lacking in the recombinant protein.

In one previously published study, a similar immunofluorescence method was used to detect anti‐cN1A autoantibodies. The specificity reported in this study was 92%, which is comparable to the specificity observed in our assay, 93% [10]. Although different serum sample collections were used in both studies, our assay seemed to be more sensitive (59%) than that of Tawara and coworkers (36%). This difference might be explained by the different cell lines used. Tawara and coworkers used monkey COS7 cells, whereas the human HEp‐2 cell line was used in our study. Also, the different secondary antibody, anti‐IgA, ‐IgG, and ‐IgM versus IgG only, may have contributed to a higher sensitivity in our assay.

The observed sensitivity and specificity of the cell‐based assay suggest that this approach for anti‐cN1A detection is superior to all other in‐house and commercial assays that have been applied previously. Further analyses with other IBM cohorts and a direct comparison with a commercially available anti‐cN1A test will be required to substantiate this.

We conclude that we have developed a cell‐based immunofluorescence assay for the detection of anti‐cN1A autoantibodies, which seems to include antibodies that target epitopes presented only in a cellular context. Our results strongly suggest that this assay is more sensitive than cN1A protein/peptide ELISA assays.

## Funding

The study was supported by the Institute for Molecules and Materials of the Radboud University, Nijmegen, the Netherlands.

## Conflicts of Interest

Ger J. M. Pruijn is inventor of a patent (EP20120740236) licensed to Euroimmun AG.

## Supporting information




**Supporting File**: eji70181‐sup‐0001‐SuppMat.pdf.

## Data Availability

The data that support the findings of this study are available on request from the corresponding author.
