# Supplementary material for: A New Immunofluorescence Assay Allows the Sensitive Detection of Anti‐Cytosolic 5′‐Nucleotidase 1A Autoantibodies
Source: Eur J Immunol. 2026 Mar 29;56(4):e70181. doi: 10.1002/eji.70181 (PMC13033954; doi:10.1002/eji.70181)
Supplement: Supplementary file 1 — Supporting File: eji70181‐sup‐0001‐SuppMat.pdf. [file EJI-56-e70181-s001.pdf]

Supporting Information

A new immunofluorescence assay allows the sensitive detection of anti-cytosolic 5'-nucleotidase 1A autoantibodies

Fleur N. Brinkman, Isa G.A. Verlangen, Filine Swets, & Ger J.M. Pruijn

Materials and Methods

Patient samples

Sera from patients with IBM and other autoimmune diseases were selected for these studies [1]. Serum samples from IBM patients were collected at the Leiden University Medical Centre (provided by Umesh Badrising) and the Radboudumc in Nijmegen (n=82; average age at blood withdrawal 71 years; 60% men). None of the IBM patients showed clinical or serological features of other diseases. Normal healthy sera (NHS) (n=35; average age at blood withdrawal 49 years; 34% men) were obtained from the Sanquin Blood Supply Foundation (Nijmegen, The Netherlands). Samples from patients with SLE (n=28) and primary SjD (n=19) were obtained from the Radboudumc in Nijmegen. Samples from PM (n=60) and DM (n=34) patients were kindly provided by Hector Chinoy from the University of Manchester, UK. Written or verbal informed consent was obtained from most of the patients from whom sera were used. The study protocol was in accordance with the Helsinki Declaration, and all procedures were approved by the local ethics committees.

Plasmids for GFP-cN1A fusion protein expression and recombinant His_6-tagged cN1A production

A previously described DNA construct containing the full-length human cN1A cDNA, pENTR/TEV/D-TOPO-cN1A [2], was used to isolate the cN1A coding sequence, which was subsequently inserted into the pcDNA5/FRT/TO vector. The GFP^{A206K} sequence was inserted at the N-terminal side of the cN1A sequence. Since the physiological relevance of the A823G nucleotide substitution in the cN1A cDNA is not supported by sequence database information, this was adjusted in accordance with cN1A REFSEQ (NM_032526.1). The resulting expression plasmid (pcDNA5/FRT/TO-GFP^{A206K}-cN1A) contains the sequence encoding the dimerization-deficient GFP variant GFP^{A206K} in frame with the cN1A coding sequence [3]. The full-length cN1A cDNA was excised from this plasmid by digestion with NotI and XhoI and inserted into a eukaryotic expression vector (pIRES-EGFP-puro; Addgene: #45567), allowing the simultaneous expression of C-terminally His_6-tagged cN1A and, in parallel, GFP, the translation of which is mediated by an IRES (pIRES-cN1A-His_6-GFP).

Cell culture

HEp-2 cells were maintained at 37 °C in a humidified incubator with 5% CO_2 in Dulbecco's modified Eagle's medium (DMEM), high glucose, GlutaMAX™-I, and pyruvate (Gibco™, Thermo Scientific), supplemented with 10% (v/v) fetal bovine serum (Sigma-Aldrich) and penicillin-streptomycin (Gibco). Cells were split twice a week to maintain cellular confluency below 90%.

Preparation of cells for immunofluorescence assay

HEp-2 cells were seeded in 6-well plates (Sarstedt) and incubated for 24 hours. Cells were transfected with pcDNA5/FRT/TO-GFP^{A206K}-cN1A using polyethylenimine and incubated for 36 hours. Then, 30,000 cells per well were seeded in transparent flat-bottom 96-well plates (Sarstedt) to provide a subconfluent monolayer. After an overnight incubation, cells were fixed with 4% paraformaldehyde. Plates were kept in PBS in the dark at 4 °C until staining.

Indirect immunofluorescence assay

All the steps of the immunostaining were carried out at room temperature. Between all incubations, wells were washed three times with PBS. After fixation, transfected HEp-2 cells were permeabilized by incubation with 0.1% Triton X-100 in PBS for 20 minutes and blocked with 5% bovine serum albumin (BSA) and 10% normal sheep serum (NSS) in PBS for one hour. Rabbit anti-cN1A (Abcam) (10 µg/ml) was used with goat-anti-rabbit-IgG-Alexa Fluor647 (Invitrogen) (4 µg/ml). Sera were diluted 50-fold and 50 µl per well was used to incubate the cells for one hour. Titers of positive sera were determined by two-fold dilution series up to 1:204800. Bound human antibodies were visualized by incubation with goat anti-human Ig-Alexa Fluor647 (Southern Biotech, 2010-31) diluted to 0.5 µg/mL in the dark. This secondary antibody reacts with the heavy and light chains of human antibodies of the IgA, IgG, and IgM isotypes. Patients' sera, primary antibody, and the secondary antibodies were diluted in 1% BSA, 10% NSS in PBS. The nuclei of cells in some samples were stained with DAPI (1 µg/mL) after both antibody incubations. Mowiol 4-88 mounting medium was added to the wells to seal the cells. Plates were kept at 4 °C in the dark until cell staining was imaged using a fluorescence microscope (EVOS M5000, Thermo Fisher Scientific), using a 40x objective (Invitrogen). Optimal microscope light settings were determined using positive and negative controls. To interpret the images, three people assessed the images blindly and independently after receiving the same instructions. Reactivity scores were – (minus) when serum antibody signals co-localizing with GFP-cN1A staining were lacking, + for low-intensity signals for the serum antibodies co-localizing with the GFP-cN1A signals, and ++ for strong serum antibody signals overlapping with the GFP-cN1A staining. Anti-cN1A titers were defined as the dilution at which the sera still showed a signal co-localizing with GFP-cN1A. The reactivity score for a given sample was assigned when two or all three evaluators gave the same score.

Western blotting

Extracts from transfected HEp-2 cells ($4*10^5$ cells) and non-transfected HEp-2 cells ($7*10^5$ cells) were separated by 12% SDS-PAGE and transferred to a nitrocellulose membrane (Amersham™ Protran®) by electroblotting. The blot was blocked with 5% non-fat dry milk in PBS containing 0.05% Tween-20 (PBST). cN1A was detected by subsequent incubations with rabbit-anti-cN1A antibodies (0.5 µg/mL, A30631, Boster) and goat-anti-rabbit IRDye800 (0.2 µg/mL, 926-32211, LI-COR Biosciences). GFP was detected by

a monoclonal mouse-anti-GFP antibody (0.2 µg/mL, F56-6A1.2.3, Invitrogen) and goat-anti-mouse IRDye680 (0.1 µg/mL926-68070, LI-COR Biosciences). Bound antibodies were visualized using the G:Box chemi XX6 (Genesys).

Protein production and purification

HEK293-6E suspension cells were maintained at less than 2 million cells per mL and kept at 37 °C in a humidified incubator with 5% *(v/v)* CO_2, shaking at 270 rpm in Freestyle F17 medium supplemented with penicillin-streptomycin, 0.1% Kolliphor P188, and GlutaMAX™-I. Cells were transfected at 1 million cells per mL with 0.5 µg/mL recombinant cN1A expression plasmid and 0.75 µl/mL FectoPRO (polyplus). After 30 min, 0.5 µl/mL of the Booster was added. Four days after transfection, cells were collected by centrifugation for 10 min at 1,000xg. Cells were washed once with PBS. The cell pellet was resuspended in lysis buffer (50 mM sodium phosphate, pH 8.0, 300 mM sodium chloride, 2% Tween-20, 50 mM imidazole, 10% glycerol, and 1x Complete proteinase inhibitor (Roche)) and sonicated for 10 cycles, 15 sec on, 15 sec off. The cell lysate was centrifuged for 10 min at 20,000x*g* at 4 °C. Ni-NTA agarose beads were washed with column buffer (50 mM sodium phosphate, pH 8.0, 300 mM sodium chloride, and 20 mM imidazole). The supernatant of the cell lysate was incubated overnight with the washed Ni-NTA agarose beads at 4 °C while rotating head-over-head. The next day, the suspension was transferred to a column. After collecting the flowthrough, the column was washed three times with 3 column volumes column buffer containing 10% glycerol, and bound proteins were eluted with elution buffer (50 mM sodium phosphate, pH 8.0, 300 mM sodium chloride, 500 mM imidazole, and 10% glycerol). A 3 kDa MWCO Millipore spin filter was used to concentrate the eluted proteins and to exchange the elution buffer for 50 mM sodium phosphate, pH 8.0, 300 mM sodium chloride, and 10% glycerol. The concentration of recombinant cN1A was determined with a BCA assay. The recombinant protein was aliquoted and stored at -20 °C.

ELISA

Nunc Maxisorp 96-well plates were coated with 50 ng recombinant cN1A in 100 µl coating buffer (0.1 M carbonate-bicarbonate buffer, pH 9.5) per well. Subsequently, wells were blocked with 1% BSA in PBS, incubated with 100-fold diluted patient serum in PBS, 1% BSA, followed by rabbit anti-human IgA, IgG, IgM, kappa, lambda-HRP (0.65 µg/ml, Dako) in PBS, 1% BSA. Between all steps, wells were washed five times with PBST using a plate washer (Nunc-Immuno Wash 8). All incubation steps were executed for 1 hour at room temperature. Bound antibodies were visualized by incubation with TMB (3,3',5,5'-tetramethylbenzidine) in phosphate citrate buffer. After 10 min, an equal volume of 2M H_2SO_4 was added to stop the reaction. The absorbance at 540 nm was subtracted from the absorbance at 450 nm (Varioskan LUX, Thermo Fisher). The cut-off values were the average values of six NHS samples plus three times the standard deviation.

References

[1] M.K. Herbert, J. Stammen-Vogelzangs, M.M. Verbeek, A. Rietveld, I.E. Lundberg, H. Chinoy, J.A. Lamb, R.G. Cooper, M. Roberts, U.A. Badrising, J.L. De Bleecker, P.M. Machado, M.G. Hanna, L. Plestilova, J. Vencovsky, B.G. Van Engelen, G.J.M. Pruijn, Disease specificity of autoantibodies to

cytosolic 5′-nucleotidase 1A in sporadic inclusion body myositis versus known autoimmune diseases, Annals of the Rheumatic Diseases 75 (2016) 696–701. https://doi.org/10.1136/annrheumdis-2014-206691.
[2] H. Pluk, B.J.A. Van Hoeve, S.H.J. Van Dooren, J. Stammen-Vogelzangs, A. Van Der Heijden, H.J. Schelhaas, M.M. Verbeek, U.A. Badrising, S. Arnardottir, K. Gheorghe, I.E. Lundberg, W.C. Boelens, B.G. Van Engelen, G.J.M. Pruijn, Autoantibodies to cytosolic 5′-nucleotidase 1A in inclusion body myositis, Annals of Neurology 73 (2013) 397–407. https://doi.org/10.1002/ana.23822.
[3] N.C. Shaner, P.A. Steinbach, R.Y. Tsien, A guide to choosing fluorescent proteins, Nat Methods 2 (2005) 905–909. https://doi.org/10.1038/nmeth819.

Author contributions

Fleur Brinkman – Conceptualization, methodology, supervision, validation, visualization, writing – original draft
Isa Verlangen – Investigation, visualization
Filine Swets – Investigation
Ger Pruijn – Conceptualization, methodology, project administration, validation, writing – review & editing

Supplementary figures

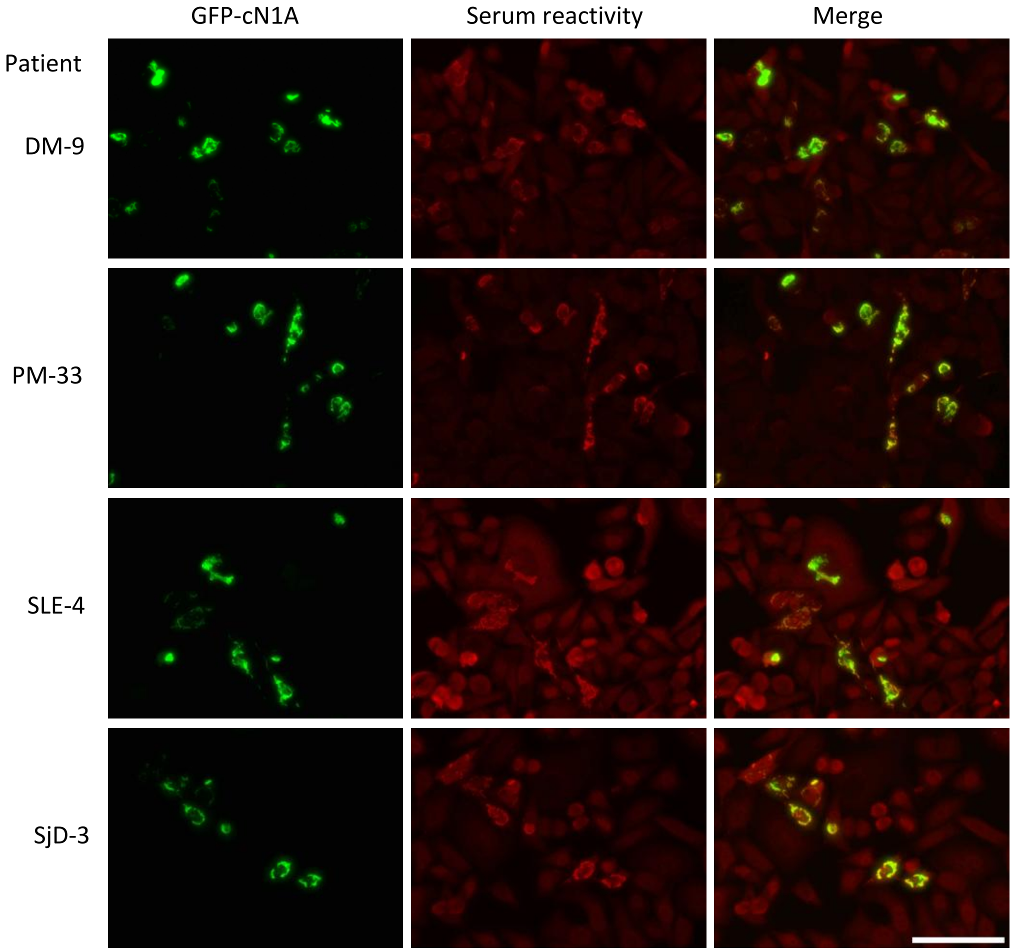

Supplementary Figure 1: Examples of images obtained with anti-cN1A-positive PM, DM, SLE, and SjD sera. Fixed, GFP-cN1A-expressing HEp-2 cells were incubated with 50-fold diluted PM, DM, SLE and SjD patient sera and bound antibodies were detected with a fluorescent secondary antibody. Green: GFP; red: serum antibody signal. Right panels: merged images. Scale bar is 100 µm.

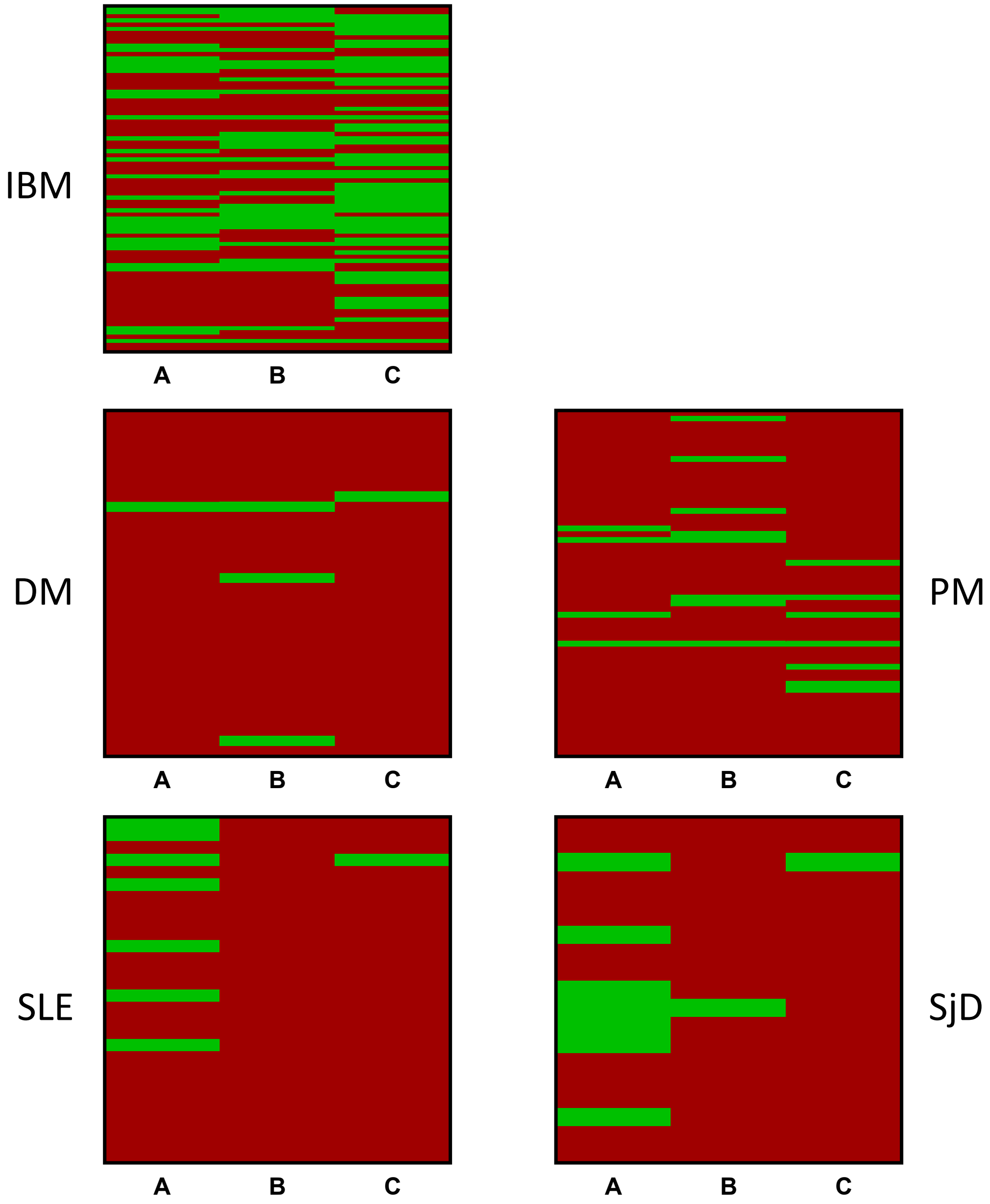

Supplementary Figure 2: Comparison of anti-cN1A reactivity of each patient serum for each anti-cN1A assay. A: peptide ELISA, B: full-length ELISA, and C: Immunofluorescence assay. Green is positive and red is negative in the anti-cN1A assay.

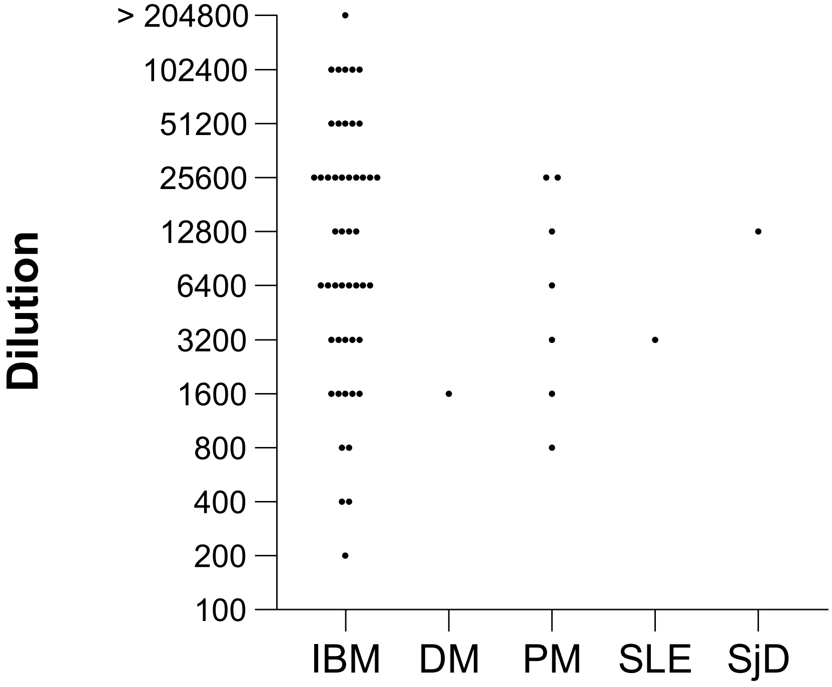

Supplementary Figure 3: Titers of anti-cN1A reactivity in positive patient samples. Titers (highest dilution that still resulted in positive anti-cN1A signals) were determined in the immunofluorescence assay by serial dilutions of sera from IBM, DM, PM, SLE and SjD patients.
